# Translation and validation of the Fatigue Severity Scale, Pittsburgh Sleep Quality Index and Modified Health Assessment Questionnaire into the Maltese Language, in a cohort of Maltese Systemic Lupus Erythematosus patients

**DOI:** 10.31138/mjr.28.4.192

**Published:** 2017-12-22

**Authors:** Rosalie Magro, Liberato Camilleri, Andrew A. Borg

**Affiliations:** 1 Rheumatology Department, Mater Dei Hospital, Msida, Malta,; 2 Statistics and Operations Research, University of Malta, Msida, Malta

**Keywords:** Self-assessment, Fatigue, Systemic Lupus Erythematosus, Sleep Quality

## Abstract

**PURPOSE:**

The assessment of fatigue, sleep quality and functional disability requires the use of validated instruments such as the Fatigue Severity Scale (FSS), the Pittsburgh Sleep Quality Index (PSQI) and the Modified Health Assessment Questionnaire (mHAQ) respectively. The aim of this study was to translate and validate these instruments into the Maltese Language.

**METHOD:**

Forward translation from the original English version into Maltese was carried out by two translators. The two versions were compiled to produce a preliminary initial Maltese translation. This was translated back into English by two other translators. This led to the development of the pre-final version of the Maltese translation, which was pilot-tested in 20 bilingual patients with systemic lupus erythematosus.

**RESULTS:**

Psychometric testing revealed good reliability of the Maltese translation of the three questionnaires. Cronbach’s alpha of the Maltese versions of the FSS, PSQI and mHAQ were 0.877, 0.859 and 0.897 respectively, showing good internal consistency. Validity of the Maltese version of the FSS was shown, since it had a significant positive correlation with visual analogue scale for fatigue (r=0.809, p<0.001).

**CONCLUSION:**

The Maltese translations were thus finalised, and could be used for clinical assessment or research purposes.

## INTRODUCTION

Fatigue is a common symptom present in several medical conditions including rheumatoid arthritis, systemic lupus erythematosus (SLE) and multiple sclerosis (MS). There is no consensus definition, but it is frequently described as the overwhelming sensation of weakness, lack of energy, or exhaustion that is usually unrelated to over-exertion and poorly relieved by rest. A number of instruments are available to assess the level of fatigue.^1^ The fatigue severity scale (FSS) is an instrument that has been developed by Krupp et al. which has been validated for the measurement of fatigue.^2^ It has been originally developed for use in SLE and MS, but has since then been used in a number of other medical conditions as well. It is a simple to use, self-reported questionnaire that consists of nine statements related to fatigue; each statement is scored on a seven-point Likert scale, depending on the level of agreement to the statement. The total score is the mean of the nine statements; the higher the score, the higher the level of fatigue.^1^ In 2007, the FSS was recommended by the Ad Hoc Committee on Systemic Lupus Erythematosus Response Criteria for Fatigue as the instrument of choice to measure fatigue in SLE, after fifteen different instruments were reviewed.^3^ The FSS has been translated and validated in a number of languages and is a widely used tool in clinical practice and research.^4–14^

One of the factors that influence fatigue is poor sleep quality. A number of instruments have been developed to assess sleep.^15^ Sleep quality can be measured by the Pittsburgh Sleep Quality Index (PSQI), which was developed by Buysse et al.^16^ This self-administered questionnaire consists of 19 items; 4 are free entry responses and the rest have 4-point Likert scale responses. These items assess 7 sleep quality components: subjective sleep quality, sleep latency, sleep duration, habitual sleep efficiency, sleep disturbances, use of sleeping medications and daytime dysfunction. A scoring algorithm is used to calculate the score for each component, ranging from 0 to 3; with a total score ranging from 0 to 21.^15^ The PSQI has been translated in a number of languages.^17–22^

Fatigue and sleep disturbances may have an impact on functional disability. The modified health assessment questionnaire (mHAQ) is a self-assessment questionnaire that measures the ability of the patient to carry out eight activities of daily living.^23^ The level of difficulty to carry out each activity is scored on a four-point Likert scale. The total score is calculated as the mean of the eight scores; the higher the score, the higher the level of functional disability. The mHAQ is an easy-to-use instrument, developed as a short version of the Health Assessment Questionnaire Disability Index, originally developed for use in rheumatoid arthritis and osteoporosis, but has since then been used in a variety of rheumatologic conditions.^24^ The mHAQ has also been translated and validated in Spanish.^25^

The FSS, PSQI and mHAQ have been validated in their original English versions.^2,16,23^ Planned research in the Maltese population on fatigue, sleep quality and functional disability in SLE, necessitates the translation and validation of the FSS, PSQI and mHAQ into the Maltese Language. This is because even though both English and Maltese are official languages in Malta, 11% of the Maltese population are unable to speak English and 26% are unable to read a newspaper article in English.^26^ Moreover, on questioning a random sample of 65 SLE patients living in Malta, 24 patients (36.9%) claimed that they felt more comfortable to fill in a questionnaire in Maltese as opposed to English. The aim of the study described in this paper, is to translate, validate and perform cross-cultural adaptation of the FSS, PSQI and mHAQ into the Maltese language. This will enable the use of the Maltese versions for the purpose of the planned research in the Maltese population.

## METHODOLOGY

The original FSS, PSQI and mHAQ questionnaires were translated and validated into the Maltese language according to the methodology recommended in the guidelines, as described in detail below.^27,28^ The study was approved by the University Research and Ethics Committee, and it was carried out between September and December 2016.

### Stage 1: Initial translation into Maltese

The original instruments were translated into the Maltese language by two translators (translators 1 and 2) whose mother language was Maltese. One of the translators had a medical background and was knowledgeable on the concepts being examined in the questionnaires. On the other hand, the other translator did not have a medical background and was not knowledgeable on the concepts being assessed in the questionnaires.

### Stage 2: Synthesis of the preliminary initial translation

The two translated versions of the instruments were then compared. The discrepancies between the two translated versions were then discussed at length. These were resolved by reaching a consensus on the best Maltese version that reflects the underlying meaning of the original English version. The preliminary initial forward translation into the Maltese Language of the FSS, PSQI and mHAQ, was thus produced.

### Stage 3: Back translation

The preliminary initial Maltese translation of the FSS, PSQI and mHAQ were then given to two other translators (translators 3 and 4) for back translation into English. English was the native language of these translators and they were totally blind to the original version. Both translators were unaware of the concepts being assessed in the questionnaires and did not have a medical background. This was done to check the validity of the forward translation into Maltese, by ensuring that the translated Maltese version reflects the same content as the original English questionnaires.

### Stage 4: Development of the pre-final version of the Maltese translation

The back translated English versions were then compared to the original English questionnaires and any discrepancies were highlighted. These discrepancies in the Maltese versions were discussed and changes were made to the Maltese translations to reflect the original English versions more accurately; thus producing the pre-final Maltese translations. This was carried out by the prinicpal researcher (who is also a health care professional) in collaboration with the four translators.

### Stage 5: Pilot testing of the pre-final version of the Maltese translation of the instruments using a sample of 20 bilingual SLE patients

Twenty bilingual SLE patients, who identify with the Maltese culture, gave informed consent to participate in the study. They were asked to fill in the pre-final version of the Maltese translation of the FSS, PSQI and mHAQ. They were also asked to fill in a Maltese translation of the visual analogue scale (VAS) for fatigue. The individuals were interviewed after filling in the questionnaires to evaluate the clarity of the instructions, items and response format of the questionnaires.

### Stage 6: Psychometric testing of the pre-final version of the Maltese translation in the target population (Maltese SLE patients) with a bilingual sample

The twenty bilingual SLE patients included in stage 5, were asked to fill in the original English version of the FSS, PSQI and mHAQ, 4 to 7 days after filling in the Maltese versions. Psychometric testing was carried out to assess the reliability, internal consistency and validity. Reliability of the translated original English questionnaires into Maltese was tested using Kendall’s tau test for statements having an ordinal scale and Pearson’s correlation test for variables having a metric scale. For both tests, the null hypothesis specified that the reliability of the translated version was weak and was accepted if the *p* value exceeded the 0.05 level of significance. The alternative hypothesis for these tests specified that the reliability of the translated version was satisfactory and was accepted if the *p* value was less than 0.05. Internal consistency of FSS, PSQI and mHAQ was assessed using Cronbach’s alpha. Validity of the Maltese translation of the FSS was assessed by correlation of FSS with VAS for fatigue using Pearson’s correlation test. IBM SPSS statistics 24 was used to carry out the statistical tests described.

## RESULTS

The twenty bilingual SLE patients included in this study were all Caucasian and of Maltese nationality. 19 were female, and the average age was 37.7 years (range 19–58 years). **Table 1** shows the characteristics of the patients included.

**Table 1.** Table summarizing the characteristics of the twenty SLE patients included in the study. The median is given for continuous variables that are not normally distributed.

**Characteristics**	**Values**
Age, mean (S.D.) years	37.7 (10.9)
Female gender (n/N) %	19/20 (95)
Caucasian race, n/N (%)	20/20 (100)
Maltese nationality, n/N (%)	20/20 (100)
Disease duration, median (range) years	7 (2–17)
Age of SLE onset, mean (S.D.) years	29.7 (9.4)
Secondary level of education, n/N (%)	8/20 (40)
Tertiary level of education, n/N (%)	12/20 (60)
FSS, mean (S.D.)	4.82 (0.99)
VAS Fatigue, median (range)	6 (0–9)
PSQI, mean (S.D.)	7.25 (4.63)
mHAQ, median (range)	0.125 (0–1.5)

### Reliability of the translation into the Maltese Language

Reliability of the translated original English questionnaires into Maltese was tested using Kendall’s tau test for statements having an ordinal scale. **Tables 2, 3** and **4** show the Kendall’s tau values for the statements in FSS, PSQI and mHAQ that have an ordinal scale, and their respective *p* values. Pearson’s correlation test was used for variables having a metric scale. Table 5 shows the Pearson’s R values for the statements in PSQI that have a metric scale, and their respective *p* values. The *p* values obtained during reliability testing, were all less than 0.05. The alternative hypothesis was thus accepted, indicating satisfactory reliability of the translated versions.

**Table 2.** Table showing Kendall’s tau values and p values for the statements in the FSS.

**Statement**	**Kendall’s Tau value**	**p value**
1. My motivation is lower when I am fatigued.	0.507	0.006
2. Exercise brings on my fatigue.	0.697	0.000
3. I am easily fatigued.	0.309	0.047
4. Fatigue interferes with my physical functioning.	0.391	0.024
5. Fatigue causes frequent problems for me.	0.630	0.000
6. My fatigue prevents sustained physical functioning.	0.408	0.041
7. Fatigue interferes with carrying out certain duties and responsibilities.	0.502	0.000
8. Fatigue is among my most disabling symptoms.	0.400	0.029
9. Fatigue interferes with my work, family, or social life.	0.669	0.000

**Table 3.** Table showing Kendall’s tau values and p values for questions having an ordinal scale in the PSQI.

**Statement**	**Kendall’s tau value**	**p value**
5. During the past month, how often have you had trouble sleeping because you		
A. Cannot get to sleep within 30 minutes	0.711	0.000
B. Wake up in the middle of the night or early morning	0.886	0.000
C. Have to get up to use the bathroom	0.855	0.000
D. Cannot breathe comfortably	0.769	0.003
E. Cough or snore loudly	0.840	0.000
F. Feel too cold	0.551	0.001
G. Feel too hot	0.408	0.026
H. Have bad dreams	0.827	0.000
I. Have pain	0.570	0.001
J. Other reason(s), please describe, including how often you have had trouble sleeping because of this reason(s)	0.557	0.027
6. During the past month, how would you rate your sleep quality overall	0.905	0.000
7. During the past month, how often have you taken medicine (prescribed or “over the counter”) to help you sleep?	0.993	0.007
8. During the past month, how often have you had trouble staying awake while driving, eating meals, or engaging in social activity?	0.400	0.035
9. During the past month, how much of a problem has it been for you to keep up enthusiasm to get things done	0.412	0.036

**Table 4.** Table showing Kendall’s tau values and p values for the questions in mHAQ.

**Statement**	**Kendall’s tau value**	**p value**
**At this moment,** are you able to:		
1. Dress yourself, including tying shoelaces and doing buttons?	0.993	0.007
2. Get in and out of bed?	0.899	0.000
3. Lift a full cup or glass to your mouth?	1.000	0.000
4. Walk outdoors on flat ground?	0.858	0.003
5. Wash and dry your entire body?	1.000	0.000
6. Bend down to pick up clothing from the floor?	0.859	0.000
7. Turn faucets/taps on and off?	0.750	0.008
8. Get in and out of a car?	0.822	0.000

**Table 5.** Table showing Pearson’s R values and p values for questions having a metric scale in the PSQI.

**Statement**	**Pearson’s R value**	**p value**
1. When have you usually gone to bed?	0.972	0.000
2. How long (in minutes) has it taken you to fall asleep each night?	0.953	0.000
3. When have you usually gotten up in the morning?	0.977	0.000
4. How many hours of actual sleep do you get at night? (This may be different than the number of hours you spend in bed)	0.915	0.000

### Internal Consistency

The Cronbach’s alpha measures the internal consistency of a number of related statements. A result that is 0.7 or higher is considered to be an acceptable internal consistency. Cronbach’s alpha for the Maltese translation of the FSS, PSQI and mHAQ were calculated. **Table 6** shows the Cronbach’s alpha values for the questionnaires. The results show good internal consistency. The Cronbach’s alpha values obtained are comparable to those from the original publications of the English versions of the questionnaires; 0.89 for FSS, 0.83 for PSQI and 0.85 for mHAQ.^2,16,23^

**Table 6.** Cronbach’s alpha values for the Maltese translation of FSS, PSQI and mHAQ.

	**Cronbach’s Alpha**	**Number of Items**
**FSS**	0.877	9
**PSQI – 7 components**	0.827	7
**PSQI - individual items**	0.859	14
**mHAQ**	0.897	8

**Table 7.** Fatigue Severity Scale.

Aqra u mmarka numru b’ċirku.	Ma naqbel xejn			⇨	Naqbel ħafna		
1. Il-motivazzjoni tiegħi hija iktar baxxa meta nkun għajjien(a) ħafna.	1	2	3	4	5	6	7
2. L-eżerċizzju jġib fuqi għajja kbira.	1	2	3	4	5	6	7
3. Ngħajja malajr.	1	2	3	4	5	6	7
4. L-għajja ttelifni milli nagħmel xogħol fiżiku.	1	2	3	4	5	6	7
5. L-għajja ta’ spiss toħloqli problemi.	1	2	3	4	5	6	7
6. L-għajja ma tħallinix nagħmel xogħol fiżiku fit-tul.	1	2	3	4	5	6	7
7. L-għajja ttellifni milli naqdi wħud mid-dmirijiet u r-responsabbiltajiet tiegħi.	1	2	3	4	5	6	7
8. L-għajja hija fost l-iktar sintomi li jtellfuni f’ħajti.	1	2	3	4	5	6	7
9. L-għajja ttellifni f’xogħoli, mal-familja, jew fil-ħajja soċjali tiegħi.	1	2	3	4	5	6	7

**Table 8.** Pittsburgh Sleep Quality Index.

	Qatt f’dan l-aħħar xahar	Inqas minn darba fil-ġimgħa	Darba jew darbtejn fil-ġimgħa	Tlett darbiet jew iktar fil-ġimgħa
5. Matul dan l-aħħar xahar, kemm-il darba kellek diffikulta’ biex torqod minħabba li				
A. Ma tistax torqod fl-ewwel nofs siegħa				
B. Tqum f’nofs ta’ lejl jew filgħodu kmieni				
C. Ikollok tqum biex tuża l-kamra tal-banju				
D. Ma jirnexxielekx tieħu nifs komdu				
E. Tisgħol jew tonħor jgħajjat				
F. Tħoss ħafna bard				
G. Tħoss ħafna sħana				
H. Toħlom ikrah				
I. Tkun muġugħ(a)				
J. Għal xi raġuni(jiet) oħra. Jekk jogħġbok semmihom u inkludi kemm-il darba kellek diffikulta’ biex toroqd minħabba f’dawn irraġunijiet.				
6. Matul dan l-aħħar xahar, kollox ma kollox, kif tikkunsidra li rqadt?	Tajjeb ħafna	Pjuttost tajjeb	Pjuttost ħażin	Ħażin ħafna
7. Matul dan l-aħħar xahar, kemm-il darba ħadt mediċina (birriċetta jew mingħajr riċetta tat-tabib) biex tgħinek torqod?				
8. Matul dan l-aħħar xahar, kemm-il darba batejt biex tibqa mqajjem/mqajma waqt li kont qed issuq, tiekol ikla jew tissoċjalizza ma’ ħaddieħor?				
9. Matul dan l-aħħar xahar, kemm kienet diffiċli żżomm l-entużjażmu biex tagħmel dak li għandek tagħmel?	Ma kienetx problema	Problema zgħira ħafna	Pjuttost problema	Problema kbira ħafna
10. Xi ħadd jorqod fl-istess sodda jew kamra mieghek?				
add ma jorqod fl-istess sodda jew kamra miegħi			________	
Is-sieħeb/sieħba tiegħi jorqod/torqod f’kamra oħra			________	
Is-sieħeb/sieħba tiegħi jorqod/torqod fl-istess kamra imma mhux fl-istess sodda			________	
Is-sieħeb/sieħba tiegħi jorqod/torqod fl-istess sodda			________	
	Qatt f’dan l-aħħar xahar	Inqas minn darba filġimgħa	Darba jew darbtejn fil-ġimgħa	Tlett darbiet jew iktar fil-ġimgħa
Jekk hemm xi ħadd li jorqod fl-istess kamra jew sodda miegħek saqsih(a) jekk fl-aħħar xahar kont qed:				
a) Tonħor jgħajjat				
b) Ikollok waqfien twil bejn nifs u ieħor waqt li tkun rieqed/rieqda				
c) Iċċaqlaq saqajk bl-iskossi waqt li tkun rieqed/rieqda				
d) Episodji meta tkun diżorjentat jew konfuż waqt l-irqad				
e) Nuqqas ta’ kwiet ieħor f’ġismek waqt li tkun rieqed/rieqda; jekk jogħġbok iddeskrivihom				

**Table 9.** Modified Health Assessment Questionnaire.

Bħalissa, tista:	Mingħajr EBDA diffikulta’	B’xi FTIT diffikulta’	B’ĦAFNA diffikulta’	MA NISTAX nagħmilha
Tilbes waħdek, inkluż taqfel iż-żarbun u l-buttuni?				
Tidħol u tqum mis-sodda?				
Terfa kikkra jew tazza mimlija biex tixrob?				
Timxi barra fil-wita’?				
Tinħasel u tixxotta ġismek kollu?				
Titbaxxa biex tiġbor il-ħwejjeg mill-art?				
Tiftaħ u tagħlaq vit?				
Tidħol u toħrog minn karozza?				

### Validity

**Figure 1.**
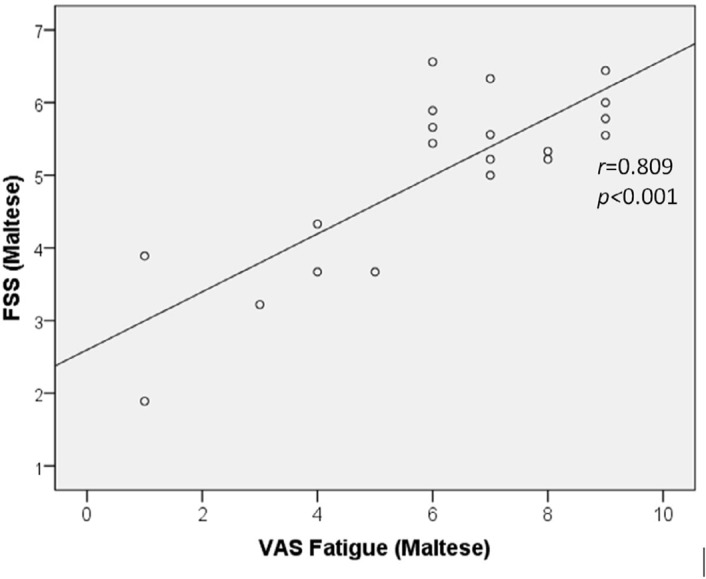
Chart showing correlation between the Maltese translation of FSS and VAS fatigue.

Validity of the Maltese translation of the FSS was assessed by correlation of FSS with visual analogue scale (VAS) for fatigue using Pearson’s correlation test. Pearson’s R value is 0.809, with a *p* value of <0.001, showing a significant positive correlation as depicted in **Figure 1**. This is concordant with the result obtained in the original publication on FSS by Krupp *et al.*, in which the R value for the correlation of FSS with VAS was 0.68 (*p*<0.001).^2^

The Maltese translations of the FSS, PSQI and mHAQ could thus be finalised as below.

## FATIGUE SEVERITY SCALE

Jekk jogħġbok indika kemm taqbel ma’ kull sentenza billi timmarka b’ċirku t-tweġiba bejn 1 u 7. Dawn jirreferu għall-ħajja normali tiegħek f’din l-aħħar ġimgħa. 1 jindika “ma naqbel xejn” u 7 jindika “naqbel ħafna”.

## PITTSBURGH SLEEP QUALITY INDEX

Il-mistoqsijiet li ġejjin huma dwar id-drawwiet talirqad tiegħek matul l-aħħar xahar biss. It-tweġibiet tiegħek għandhom jindikaw ir-risposta l-aktar eżatta għall-maġġoranza tal-jiem u l-iljieli f’dan l-aħħar xahar. Jekk jogħġbok wieġeb il-mistoqsijiet kollha.

Fl-aħħar xahar,

1. Fi x’ħin normalment dħalt torqod? _______________
2. Kemm domt (f’minuti) biex marret għajnejk bik? ____________
3. Fi x’hin normalment qomt filgħodu?___________
4. Kemm-il siegħa jirnexxielek torqod bil-lejl? (It-tweġiba tista tkun differenti minn kemm tqatta siegħat fis-sodda.) _________________

Jekk jgħoġbok immarka l-aħjar risposta:

## MODIFIED HEALTH ASSESSMENT QUESTIONNAIRE

Jekk jogħġbok immarka l-aħjar tweġiba skond l-abbiltajiet tiegħek.

## DISCUSSION

In this study, a Maltese version of the FSS, PSQI and mHAQ was developed. Psychometric testing, including assessment of reliability and internal consistency was performed in a Maltese speaking cohort of SLE patients. The results have shown adequate reliability of all the statements in the Maltese translations of the three questionnaires. Internal consistency of the Maltese versions of the FSS, PSQI and mHAQ was shown to be good, with Cronbach’s alpha values of 0.877, 0.859 and 0.897 respectively. These values are comparable to those obtained from other studies investigating the psychometric properties of these instruments. The Maltese version of the FSS had a significant positive correlation with VAS fatigue (r=0.809, p<0.001), providing evidence of its validity.

This study has a number of limitations. Even though the sample size was enough to prove statistically significant reliability, validity and internal consistency, it was relatively small. The ability of the Maltese translations of the FSS, PSQI and mHAQ, to assess changes in fatigue, sleep quality and functional disability respectively over time, was not examined. This would require further research to analyse the responsiveness of the Maltese versions of the instruments. Moreover, in this study patients with SLE as their principal diagnosis were included. In fact, it consisted of a skewed sample in which 95% were females. This is expected in a cohort of SLE patients, since females are affected nine times more frequently than males. Further research evaluating the usefulness of the Maltese translated questionnaires in other conditions and in the general population is required.

In conclusion, the assessment of the presence and severity of fatigue, poor sleep quality and functional disability requires the use of validated instruments. The Maltese version of the FSS, PSQI and mHAQ developed in this study showed adequate reliability and internal consistency in a cohort of SLE patients. They have thus been validated and can be used in clinical practice and research.

## ABBREVIATIONS

FSS: Fatigue Severity Scale
mHAQ: Modified Health Assessment Questionnaire
MS: Multiple Sclerosis
PSQI: Pittsburgh Sleep Quality Index
SLE: Systemic Lupus Erythematosus
VAS: Visual analogue scale

## References

[1] Neuberger G B. Measures of Fatigue. Arthritis Rheum 2003;49:S175–83.

[2] Krupp L B LaRocca N G Muir-Nash J Steinberg A D. The Fatigue Severity Scale: application to patients with multiple sclerosis and systemic lupus erythematosus. Arch Neurol 1989;46:1121–3. 2803071 10.1001/archneur.1989.00520460115022

[3] Ad Hoc Committee on Systemic Lupus Erythematosus Response Criteria for Fatigue Measurement of fatigue in systemic lupus erythematosus: a systematic review. Arthritis Rheum 2007;57:1348–57. 18050225 10.1002/art.23113

[4] Al-Sobayel H I Al-Hugail H A AlSaif R M Albawardi N M Alnahdi A H Daif A M Validation of an Arabic version of Fatigue Severity Scale. Saudi Med J 2016;37:73–8. 26739978 10.15537/smj.2016.1.13055 PMC4724683

[5] Azimian M Farahani A S Dadkhah A Fallahpour M Karimlu M. Fatigue severity scale: the psychometric properties of the persian-version in patients with multiple sclerosis. Res J Biol Sci 2009;4:974–7.

[6] Bakalidou D Skordilis E K Giannopoulos S Stamboulis E Voumvourakis K. Validity and reliability of the FSS in Greek MS patients. SpringerPlus 2013;2:304. 23888275 10.1186/2193-1801-2-304 PMC3710409

[7] Fereshtehnejad S M Hadizadeh H Farhadi F Shahidi G A Delbari A Lökk J. Reliability and validity of the Persian version of the fatigue severity scale in idiopathic Parkinson’s disease patients. Parkinsons Dis 2013;2013:935429. 24089644 10.1155/2013/935429 PMC3780699

[8] Gencay-Can A Can S S. Validation of the Turkish version of the fatigue severity scale in patients with fibromyalgia. Rheumatol Int 2012;32:27–31. 20658235 10.1007/s00296-010-1558-3

[9] Laranjeira C A. Translation and adaptation of the fatigue severity scale for use in Portugal. Appl Nurs Res 2012;25:212–7. 22698652 10.1016/j.apnr.2010.11.001

[10] Lerdal A Wahl A Rustøen T Hanestad B R Moum T. Fatigue in the general population: a translation and test of the psychometric properties of the Norwegian version of the fatigue severity scale. Scand J Soc Med 2005;33:123–30. 10.1080/14034940410028406 15823973

[11] Lorentzen K Danielsen M A Kay S D Voss A. Validation of the Fatigue Severity Scale in Danish patients with systemic lupus erythematosus. Dan Med J 2014;61:A4808. 24814589

[12] Mattsson M Möller B Lundberg I E Gard G Boström C. Reliability and validity of the Fatigue Severity Scale in Swedish for patients with systemic lupus erythematosus. Scand J Rheumatol 2008;37:269–77. 18612927 10.1080/03009740801914868

[13] Valderramas S Feres A C Melo A. Reliability and validity study of a Brazilian-Portuguese version of the fatigue severity scale in Parkinson’s disease patients. Arq Neuropsiquiatr 2012;70:497–500. 22836454 10.1590/s0004-282x2012000700005

[14] Valko P O Bassetti C L Bloch K E Held U Baumann C R. Validation of the fatigue severity scale in a Swiss cohort. Sleep 2008;31:1601–7. 19014080 10.1093/sleep/31.11.1601 PMC2579971

[15] Omachi T A. Measures of sleep in rheumatologic diseases: Epworth Sleepiness Scale (ESS), Functional Outcome of Sleep Questionnaire (FOSQ), Insomnia Severity Index (ISI), and Pittsburgh Sleep Quality Index (PSQI). Arthritis Care Res (Hoboken) 2011;63:S287–96. 22588751 10.1002/acr.20544 PMC3711174

[16] Buysse D J Reynolds C F 3rd Monk T H Berman S R Kupfer D J. The Pittsburgh Sleep Quality Index: a new instrument for psychiatric practice and research. Psychiatry Res 1989;28:193–213. 2748771 10.1016/0165-1781(89)90047-4

[17] Anandakumar D Dayabandara M Ratnatunga S S Hanwella R de Silva V A. Validation of the Sinhala version of the Pittsburgh Sleep Quality Index. Ceylon Med J 2016;61:22–5. 27031975 10.4038/cmj.v61i1.8255

[18] Sitasuwan T Bussaratid S Ruttanaumpawan P Chotinaiwattarakul W. Reliability and validity of the Thai version of the Pittsburgh Sleep Quality Index. J Med Assoc Thai 2014;97:S57–67. 24772581

[19] Hashmi A M Khawaja I S Butt Z Umair M Naqvi S H Jawad-Ul-Haq The Pittsburgh Sleep Quality Index: validation of the Urdu translation. J Coll Physicians Surg Pak. 2014;24:123–6. 24491008

[20] Farrahi Moghaddam J Nakhaee N Sheibani V Garrusi B Amirkafi A. Reliability and validity of the Persian version of the Pittsburgh Sleep Quality Index (PSQI-P). Sleep Breath 2012;16:79–82. 21614577 10.1007/s11325-010-0478-5

[21] Bertolazi A N Fagondes S C Hoff L S Dartora E G Miozzo I C de Barba M E Barreto S S. Validation of the Brazilian Portuguese version of the Pittsburgh Sleep Quality Index. Sleep Med 2011;12:70–5. 21145786 10.1016/j.sleep.2010.04.020

[22] Suleiman K H Yates B C Berger A M Pozehl B Meza J. Translating the Pittsburgh Sleep Quality Index into Arabic. West J Nurs Res 2010;32:250–68. 19915205 10.1177/0193945909348230

[23] Pincus T Summey J A Soraci S A Jr. Wallston K A Hummon N P. Assessment of patient satisfaction in activities of daily living using a modified Stanford Health Assessment Questionnaire. Arthritis Rheum 1983;26:1346–53. 6639693 10.1002/art.1780261107

[24] Fries J F Spitz P Kraines R G Holman H R. Measurement of patient outcome in arthritis. Arthritis Rheum 1980;23:137–45. 7362664 10.1002/art.1780230202

[25] González V Stewart A Ritter P Lorig K. Translation and validation of arthritis outcome measures into Spanish. Arthritis Rheum 1995;38:1429–46. 7575693 10.1002/art.1780381010

[26] European Commission. Special Eurobarometer 386: Europeans and Their Languages. 2012 http://ec.europa.eu/commfrontoffice/publicopinion/archives/ebs/ebs_386_en.pdf

[27] Beaton D E Bombardier C Guillemin F Ferraz M B. Guidelines for the process of cross-cultural adaptation of self-report measures. Spine 2000;25:3186–91. 11124735 10.1097/00007632-200012150-00014

[28] Sousa V D Rojjanasrirat W. Translation, adaptation and validation of instruments or scales for use in cross-cultural health care research: a clear and user-friendly guideline. J Eval Clin Pract 2011;17:268–74. 20874835 10.1111/j.1365-2753.2010.01434.x

